# Supplementary material for: Association between oral microbiome and seven types of cancers in East Asian population: a two-sample Mendelian randomization analysis
Source: Front Mol Biosci. 2023 Nov 21;10:1327893. doi: 10.3389/fmolb.2023.1327893 (PMC10702768; doi:10.3389/fmolb.2023.1327893)
Supplement: Supplementary file 1 [file DataSheet1.ZIP › Supplementary Materials/MR plots of saliva/saliva═╝/Lung Cancer/pheno.2886_to_lung cancer_leave_one_out.pdf]

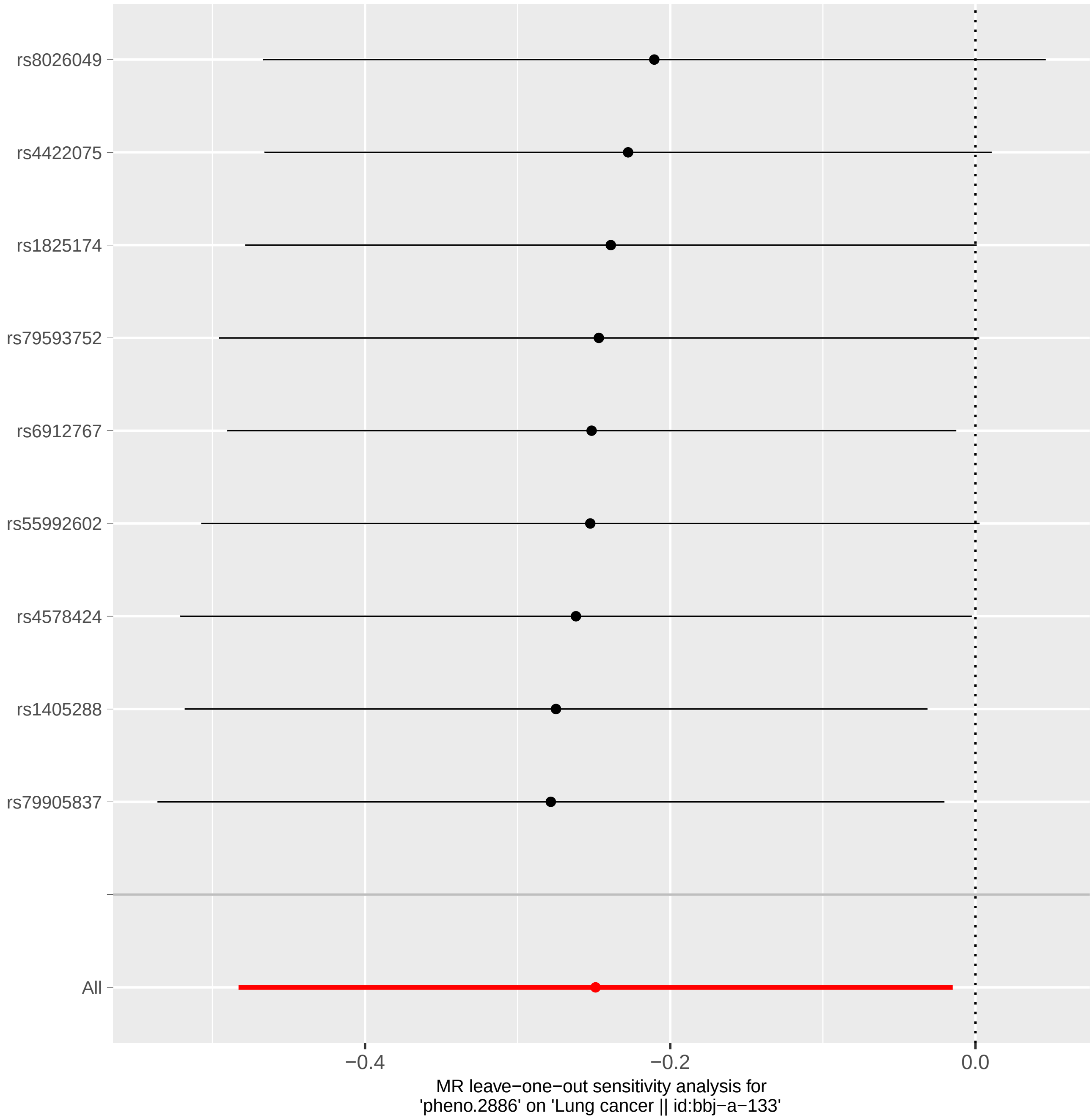
rs8026049
rs4422075
rs1825174
rs79593752
rs6912767
rs55992602
rs4578424
rs1405288
rs79905837
All
−0.4
−0.2
0.0
MR leave−one−out sensitivity analysis for
'pheno.2886' on 'Lung cancer || id:bbj−a−133'
